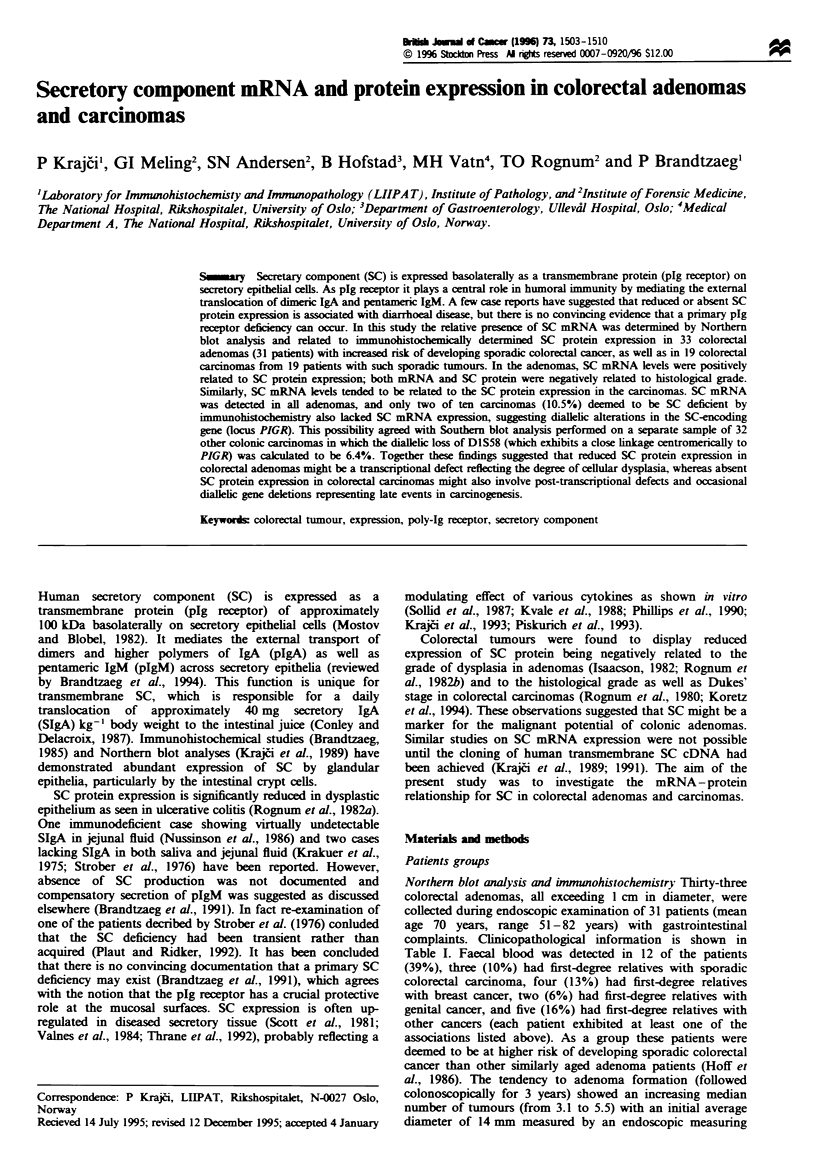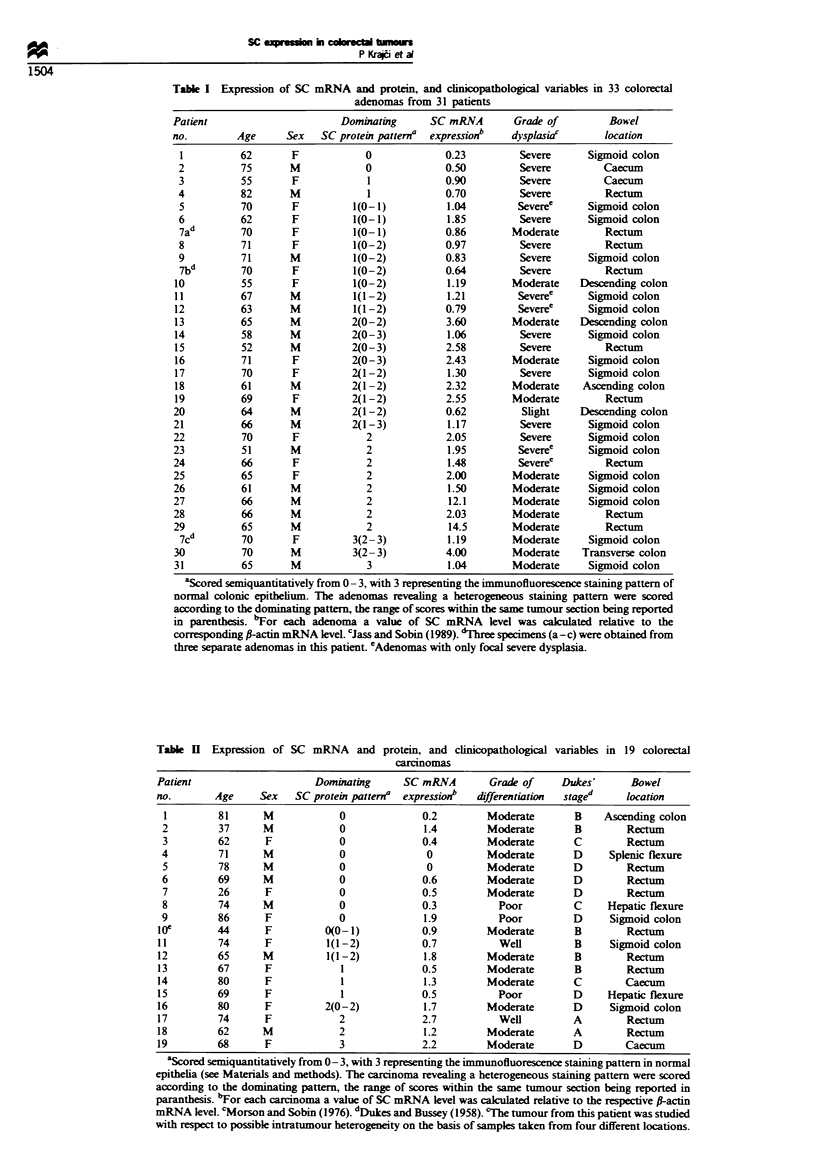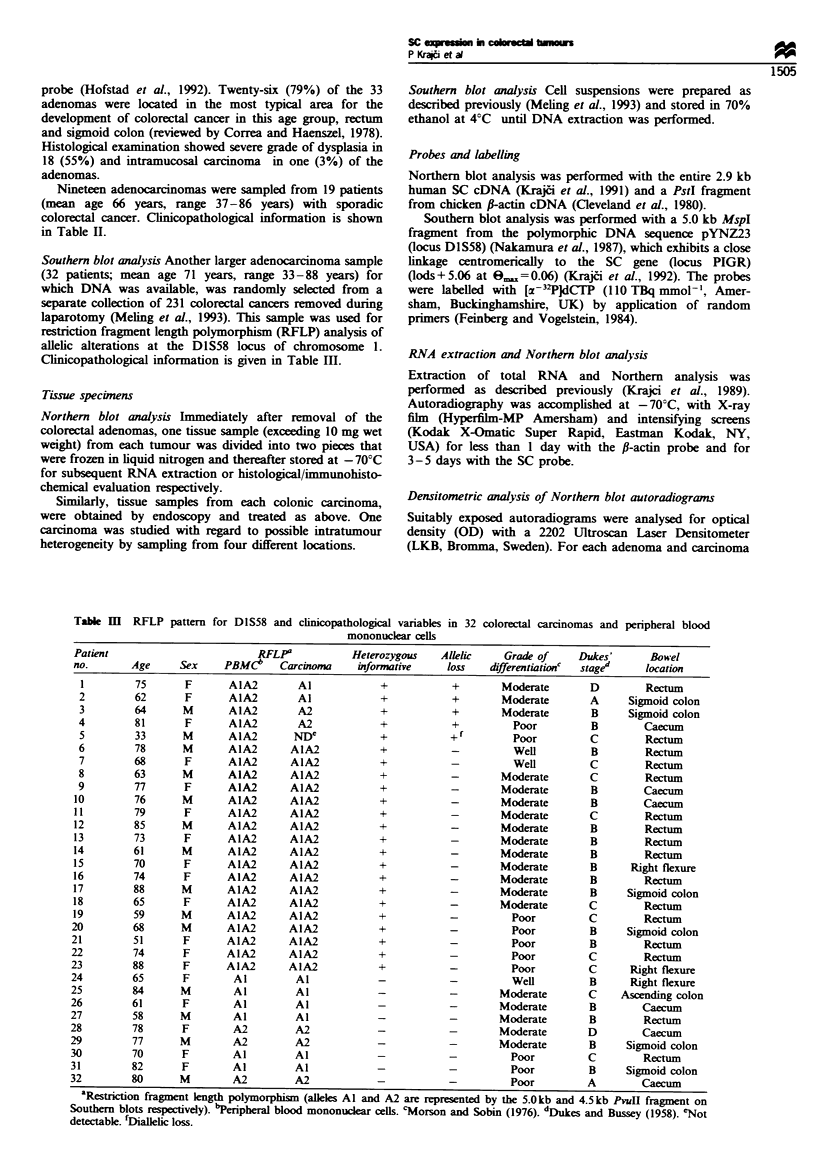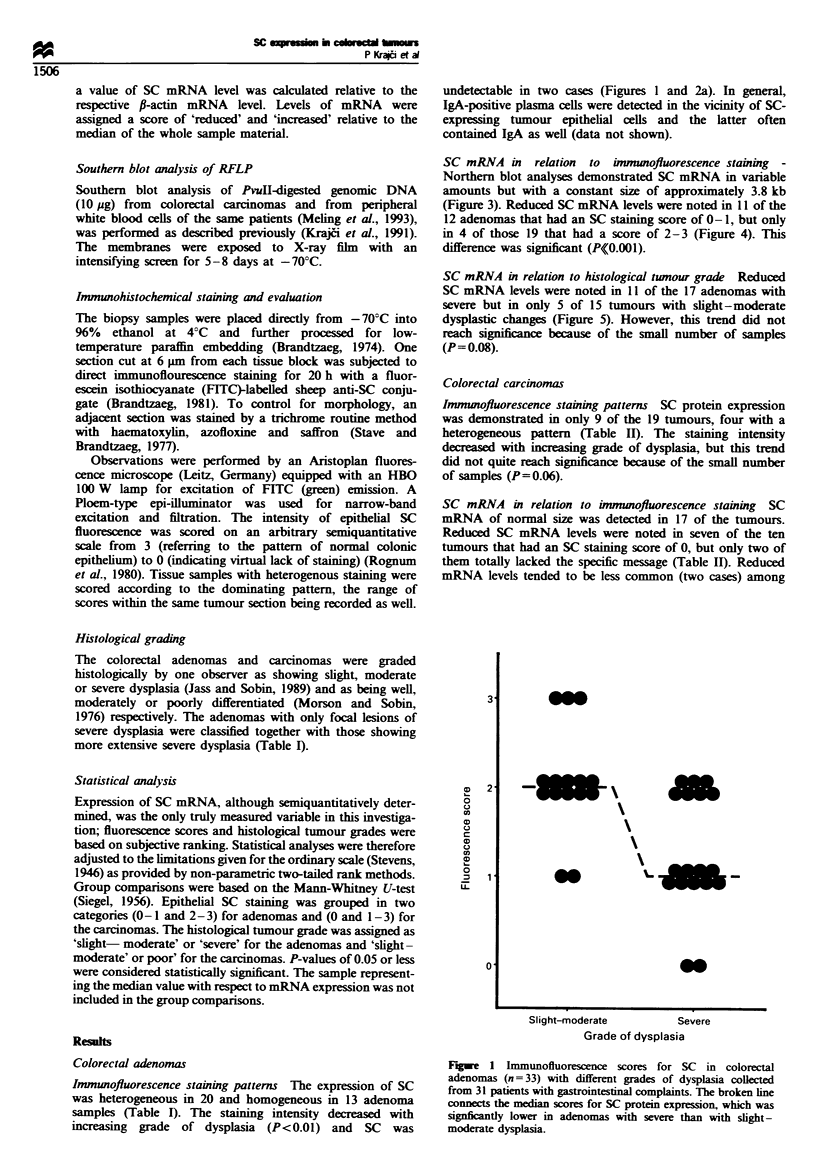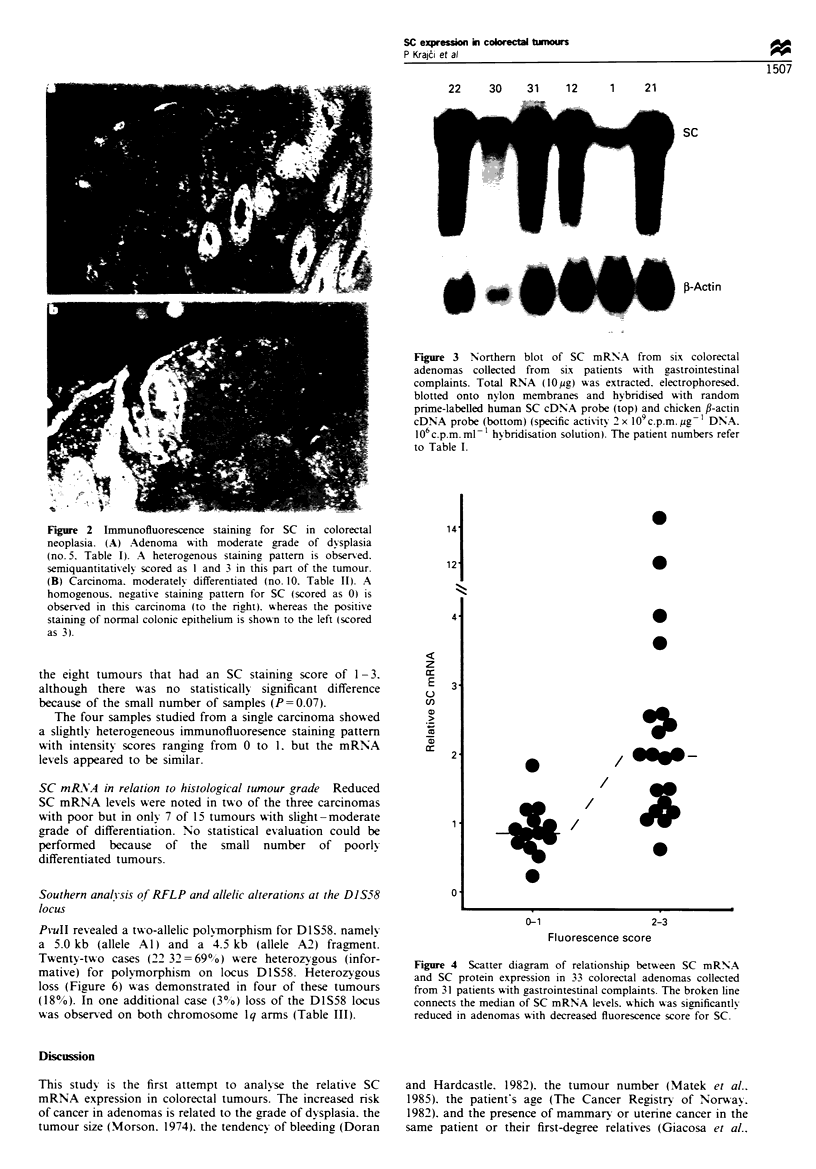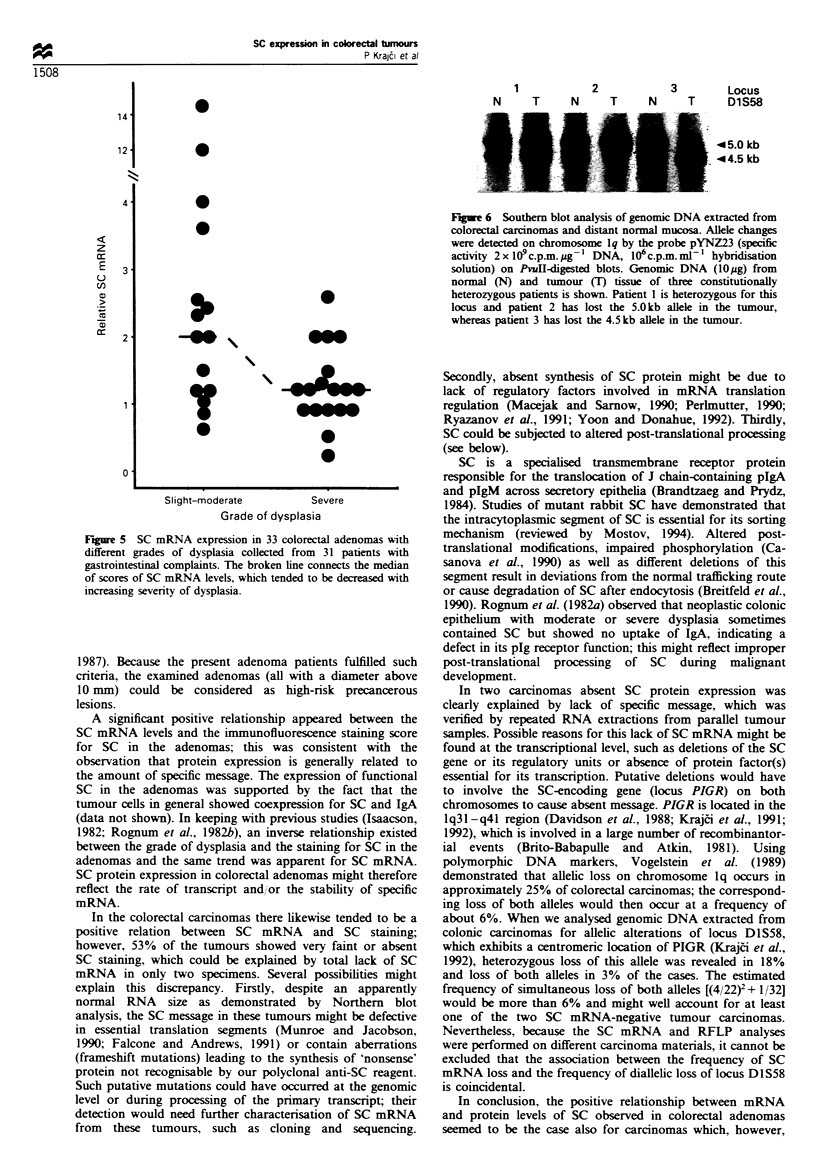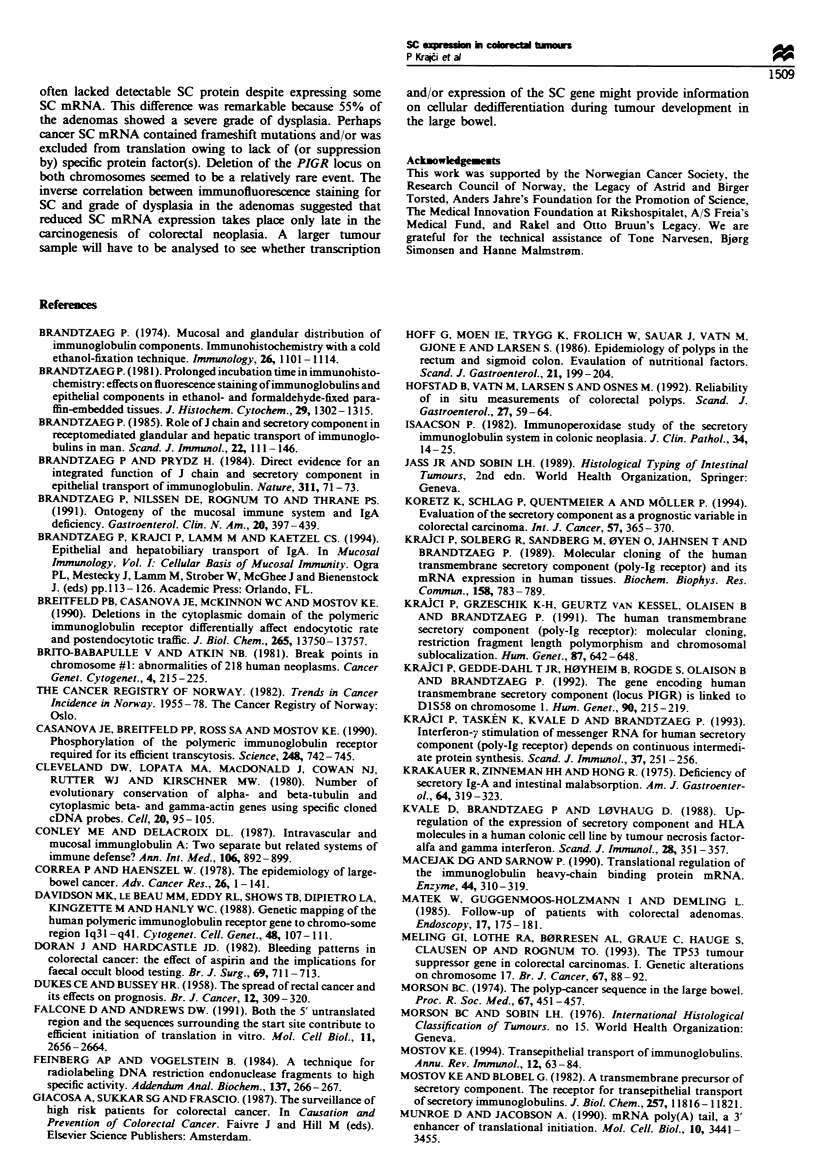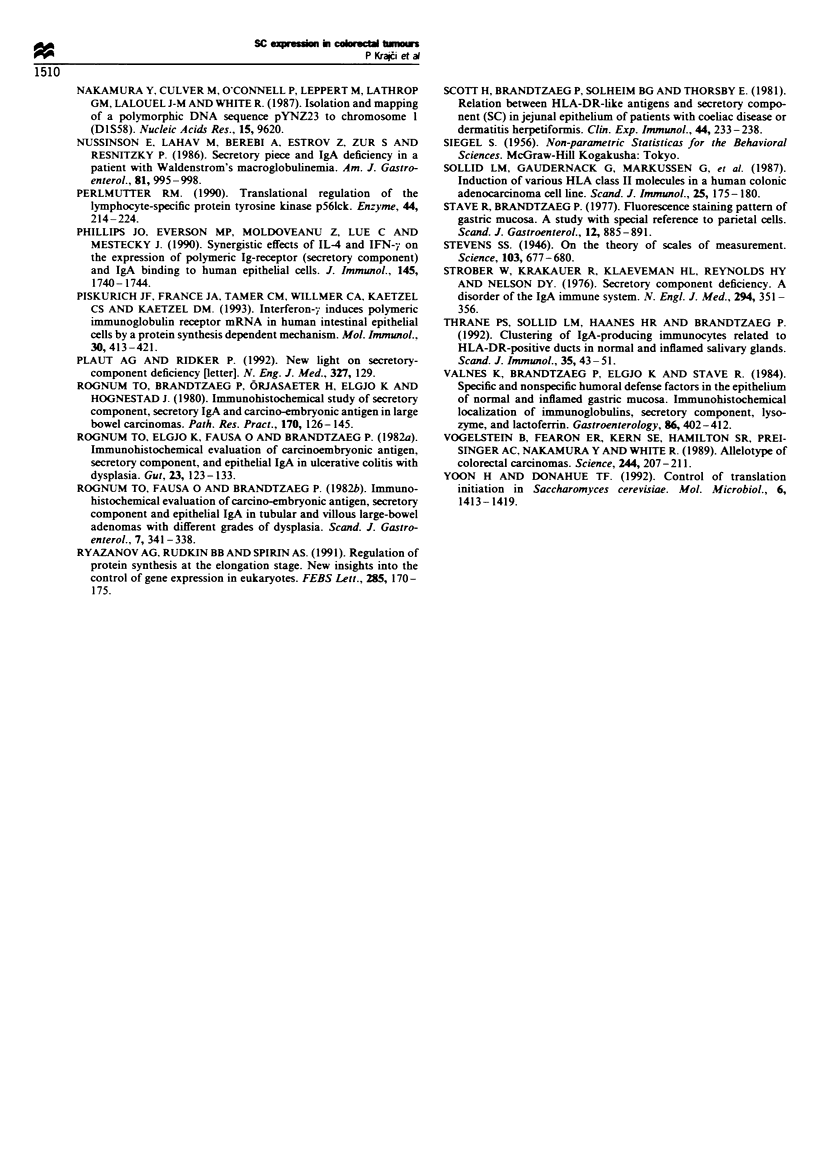# Secretory component mRNA and protein expression in colorectal adenomas and carcinomas.

**DOI:** 10.1038/bjc.1996.284

**Published:** 1996-06

**Authors:** P. Krajci, G. I. Meling, S. N. Andersen, B. Hofstad, M. H. Vatn, T. O. Rognum, P. Brandtzaeg

**Affiliations:** Laboratory for Immunohistochemisty and Immunopathology (LIIPAT), Institute of Pathology, Rikshospitalet, University of Oslo, Norway.

## Abstract

**Images:**


© 1996 Stockton Press All rights reserved 0007-0920/96 $12.00

### Secretory component mRNA and protein expression in colorectal adenomas and carcinomas

P Krajči[1], GI Meling[2], SN Andersen[2], B Hofstad[3], MH Vatn[4], TO Rognum[2] and P Brandtzaeg[1]

[1]*Laboratory for Immunohistochemisty and Immunopathology (LIIPAT), Institute of Pathology, and* [2]*Institute of Forensic Medicine, The National Hospital, Rikshospitalet, University of Oslo;* [3]*Department of Gastroenterology, Ullevål Hospital, Oslo;* [4]*Medical Department A, The National Hospital, Rikshospitalet, University of Oslo, Norway.*

**Summary** Secretary component (SC) is expressed basolaterally as a transmembrane protein (pIg receptor) on secretory epithelial cells. As pIg receptor it plays a central role in humoral immunity by mediating the external translocation of dimeric IgA and pentameric IgM. A few case reports have suggested that reduced or absent SC protein expression is associated with diarrhoeal disease, but there is no convincing evidence that a primary pIg receptor deficiency can occur. In this study the relative presence of SC mRNA was determined by Northern blot analysis and related to immunohistochemically determined SC protein expression in 33 colorectal adenomas (31 patients) with increased risk of developing sporadic colorectal cancer, as well as in 19 colorectal carcinomas from 19 patients with such sporadic tumours. In the adenomas, SC mRNA levels were positively related to SC protein expression; both mRNA and SC protein were negatively related to histological grade. Similarly, SC mRNA levels tended to be related to the SC protein expression in the carcinomas. SC mRNA was detected in all adenomas, and only two of ten carcinomas (10.5%) deemed to be SC deficient by immunohistochemistry also lacked SC mRNA expression, suggesting diallelic alterations in the SC-encoding gene (locus *PIGR*). This possibility agreed with Southern blot analysis performed on a separate sample of 32 other colonic carcinomas in which the diallelic loss of D1S58 (which exhibits a close linkage centromerically to *PIGR*) was calculated to be 6.4%. Together these findings suggested that reduced SC protein expression in colorectal adenomas might be a transcriptional defect reflecting the degree of cellular dysplasia, whereas absent SC protein expression in colorectal carcinomas might also involve post-transcriptional defects and occasional diallelic gene deletions representing late events in carcinogenesis.

**Keywords:** colorectal tumour, expression, poly-Ig receptor, secretory component

Human secretory component (SC) is expressed as a transmembrane protein (pIg receptor) of approximately 100 kDa basolaterally on secretory epithelial cells (Mostov and Blobel, 1982). It mediates the external transport of dimers and higher polymers of IgA (pIgA) as well as pentameric IgM (pIgM) across secretory epithelia (reviewed by Brandtzaeg *et al.*, 1994). This function is unique for transmembrane SC, which is responsible for a daily translocation of approximately 40 mg secretory IgA (SIgA) $kg^{-1}$ body weight to the intestinal juice (Conley and Delacroix, 1987). Immunohistochemical studies (Brandtzaeg, 1985) and Northern blot analyses (Krajči *et al.*, 1989) have demonstrated abundant expression of SC by glandular epithelia, particularly by the intestinal crypt cells.

SC protein expression is significantly reduced in dysplastic epithelium as seen in ulcerative colitis (Rognum *et al.*, 1982*a*). One immunodeficient case showing virtually undetectable SIgA in jejunal fluid (Nussinson *et al.*, 1986) and two cases lacking SIgA in both saliva and jejunal fluid (Krakuer *et al.*, 1975; Strober *et al.*, 1976) have been reported. However, absence of SC production was not documented and compensatory secretion of pIgM was suggested as discussed elsewhere (Brandtzaeg *et al.*, 1991). In fact re-examination of one of the patients decribed by Strober *et al.* (1976) conluded that the SC deficiency had been transient rather than acquired (Plaut and Ridker, 1992). It has been concluded that there is no convincing documentation that a primary SC deficiency may exist (Brandtzaeg *et al.*, 1991), which agrees with the notion that the pIg receptor has a crucial protective role at the mucosal surfaces. SC expression is often up-regulated in diseased secretory tissue (Scott *et al.*, 1981; Valnes *et al.*, 1984; Thrane *et al.*, 1992), probably reflecting a modulating effect of various cytokines as shown *in vitro* (Sollid *et al.*, 1987; Kvale *et al.*, 1988; Phillips *et al.*, 1990; Krajči *et al.*, 1993; Piskurich *et al.*, 1993).

Colorectal tumours were found to display reduced expression of SC protein being negatively related to the grade of dysplasia in adenomas (Isaacson, 1982; Rognum *et al.*, 1982*b*) and to the histological grade as well as Dukes' stage in colorectal carcinomas (Rognum *et al.*, 1980; Koretz *et al.*, 1994). These observations suggested that SC might be a marker for the malignant potential of colonic adenomas. Similar studies on SC mRNA expression were not possible until the cloning of human transmembrane SC cDNA had been achieved (Krajči *et al.*, 1989; 1991). The aim of the present study was to investigate the mRNA–protein relationship for SC in colorectal adenomas and carcinomas.

### Materials and methods

### *Patients groups*

*Northern blot analysis and immunohistochemistry* Thirty-three colorectal adenomas, all exceeding 1 cm in diameter, were collected during endoscopic examination of 31 patients (mean age 70 years, range 51–82 years) with gastrointestinal complaints. Clinicopathological information is shown in Table I. Faecal blood was detected in 12 of the patients (39%), three (10%) had first-degree relatives with sporadic colorectal carcinoma, four (13%) had first-degree relatives with breast cancer, two (6%) had first-degree relatives with genital cancer, and five (16%) had first-degree relatives with other cancers (each patient exhibited at least one of the associations listed above). As a group these patients were deemed to be at higher risk of developing sporadic colorectal cancer than other similarly aged adenoma patients (Hoff *et al.*, 1986). The tendency to adenoma formation (followed colonoscopically for 3 years) showed an increasing median number of tumours (from 3.1 to 5.5) with an initial average diameter of 14 mm measured by an endoscopic measuring

Correspondence: P Krajči, LIIPAT, Rikshospitalet, N-0027 Oslo, Norway
Recieved 14 July 1995; revised 12 December 1995; accepted 4 January

**Table I** Expression of SC mRNA and protein, and clinicopathological variables in 33 colorectal adenomas from 31 patients

| *Patient no.* | *Age* | *Sex* | *Dominating SC protein pattern*[a] | *SC mRNA expression*[b] | *Grade of dysplasia*[c] | *Bowel location* |
|---|---|---|---|---|---|---|
| 1 | 62 | F | 0 | 0.23 | Severe | Sigmoid colon |
| 2 | 75 | M | 0 | 0.50 | Severe | Caecum |
| 3 | 55 | F | 1 | 0.90 | Severe | Caecum |
| 4 | 82 | M | 1 | 0.70 | Severe | Rectum |
| 5 | 70 | F | 1(0-1) | 1.04 | Severe[e] | Sigmoid colon |
| 6 | 62 | F | 1(0-1) | 1.85 | Severe | Sigmoid colon |
| 7a[d] | 70 | F | 1(0-1) | 0.86 | Moderate | Rectum |
| 8 | 71 | F | 1(0-2) | 0.97 | Severe | Rectum |
| 9 | 71 | M | 1(0-2) | 0.83 | Severe | Sigmoid colon |
| 7b[d] | 70 | F | 1(0-2) | 0.64 | Severe | Rectum |
| 10 | 55 | F | 1(0-2) | 1.19 | Moderate | Descending colon |
| 11 | 67 | M | 1(1-2) | 1.21 | Severe[e] | Sigmoid colon |
| 12 | 63 | M | 1(1-2) | 0.79 | Severe[e] | Sigmoid colon |
| 13 | 65 | M | 2(0-2) | 3.60 | Moderate | Descending colon |
| 14 | 58 | M | 2(0-3) | 1.06 | Severe | Sigmoid colon |
| 15 | 52 | M | 2(0-3) | 2.58 | Severe | Rectum |
| 16 | 71 | F | 2(0-3) | 2.43 | Moderate | Sigmoid colon |
| 17 | 70 | F | 2(1-2) | 1.30 | Severe | Sigmoid colon |
| 18 | 61 | M | 2(1-2) | 2.32 | Moderate | Ascending colon |
| 19 | 69 | F | 2(1-2) | 2.55 | Moderate | Rectum |
| 20 | 64 | M | 2(1-2) | 0.62 | Slight | Descending colon |
| 21 | 66 | M | 2(1-3) | 1.17 | Severe | Sigmoid colon |
| 22 | 70 | F | 2 | 2.05 | Severe | Sigmoid colon |
| 23 | 51 | M | 2 | 1.95 | Severe[e] | Sigmoid colon |
| 24 | 66 | F | 2 | 1.48 | Severe[e] | Rectum |
| 25 | 65 | F | 2 | 2.00 | Moderate | Sigmoid colon |
| 26 | 61 | M | 2 | 1.50 | Moderate | Sigmoid colon |
| 27 | 66 | M | 2 | 12.1 | Moderate | Sigmoid colon |
| 28 | 66 | M | 2 | 2.03 | Moderate | Rectum |
| 29 | 65 | M | 2 | 14.5 | Moderate | Rectum |
| 7c[d] | 70 | F | 3(2-3) | 1.19 | Moderate | Sigmoid colon |
| 30 | 70 | M | 3(2-3) | 4.00 | Moderate | Transverse colon |
| 31 | 65 | M | 3 | 1.04 | Moderate | Sigmoid colon |

[a]Scored semiquantitatively from 0-3, with 3 representing the immunofluorescence staining pattern of normal colonic epithelium. The adenomas revealing a heterogeneous staining pattern were scored according to the dominating pattern, the range of scores within the same tumour section being reported in parenthesis. [b]For each adenoma a value of SC mRNA level was calculated relative to the corresponding $\beta$-actin mRNA level. [c]Jass and Sobin (1989). [d]Three specimens (a-c) were obtained from three separate adenomas in this patient. [e]Adenomas with only focal severe dysplasia.

**Table II** Expression of SC mRNA and protein, and clinicopathological variables in 19 colorectal carcinomas

| *Patient no.* | *Age* | *Sex* | *Dominating SC protein pattern*[a] | *SC mRNA expression*[b] | *Grade of differentiation* | *Dukes' stage*[d] | *Bowel location* |
|---|---|---|---|---|---|---|---|
| 1 | 81 | M | 0 | 0.2 | Moderate | B | Ascending colon |
| 2 | 37 | M | 0 | 1.4 | Moderate | B | Rectum |
| 3 | 62 | F | 0 | 0.4 | Moderate | C | Rectum |
| 4 | 71 | M | 0 | 0 | Moderate | D | Splenic flexure |
| 5 | 78 | M | 0 | 0 | Moderate | D | Rectum |
| 6 | 69 | M | 0 | 0.6 | Moderate | D | Rectum |
| 7 | 26 | F | 0 | 0.5 | Moderate | D | Rectum |
| 8 | 74 | M | 0 | 0.3 | Poor | C | Hepatic flexure |
| 9 | 86 | F | 0 | 1.9 | Poor | D | Sigmoid colon |
| 10[e] | 44 | F | 0(0-1) | 0.9 | Moderate | B | Rectum |
| 11 | 74 | F | 1(1-2) | 0.7 | Well | B | Sigmoid colon |
| 12 | 65 | M | 1(1-2) | 1.8 | Moderate | B | Rectum |
| 13 | 67 | F | 1 | 0.5 | Moderate | B | Rectum |
| 14 | 80 | F | 1 | 1.3 | Moderate | C | Caecum |
| 15 | 69 | F | 1 | 0.5 | Poor | D | Hepatic flexure |
| 16 | 80 | F | 2(0-2) | 1.7 | Moderate | D | Sigmoid colon |
| 17 | 74 | F | 2 | 2.7 | Well | A | Rectum |
| 18 | 62 | M | 2 | 1.2 | Moderate | A | Rectum |
| 19 | 68 | F | 3 | 2.2 | Moderate | D | Caecum |

[a]Scored semiquantitatively from 0-3, with 3 representing the immunofluorescence staining pattern in normal epithelia (see Materials and methods). The carcinoma revealing a heterogeneous staining pattern were scored according to the dominating pattern, the range of scores within the same tumour section being reported in paranthesis. [b]For each carcinoma a value of SC mRNA level was calculated relative to the respective $\beta$-actin mRNA level. [c]Morson and Sobin (1976). [d]Dukes and Bussey (1958). [e]The tumour from this patient was studied with respect to possible intratumour heterogeneity on the basis of samples taken from four different locations.

probe (Hofstad *et al.*, 1992). Twenty-six (79%) of the 33 adenomas were located in the most typical area for the development of colorectal cancer in this age group, rectum and sigmoid colon (reviewed by Correa and Haenszel, 1978). Histological examination showed severe grade of dysplasia in 18 (55%) and intramucosal carcinoma in one (3%) of the adenomas.

Nineteen adenocarcinomas were sampled from 19 patients (mean age 66 years, range 37–86 years) with sporadic colorectal cancer. Clinicopathological information is shown in Table II.

*Southern blot analysis* Another larger adenocarcinoma sample (32 patients; mean age 71 years, range 33–88 years) for which DNA was available, was randomly selected from a separate collection of 231 colorectal cancers removed during laparotomy (Meling *et al.*, 1993). This sample was used for restriction fragment length polymorphism (RFLP) analysis of allelic alterations at the D1S58 locus of chromosome 1. Clinicopathological information is given in Table III.

### *Tissue specimens*

*Northern blot analysis* Immediately after removal of the colorectal adenomas, one tissue sample (exceeding 10 mg wet weight) from each tumour was divided into two pieces that were frozen in liquid nitrogen and thereafter stored at −70°C for subsequent RNA extraction or histological/immunohistochemical evaluation respectively.

Similarly, tissue samples from each colonic carcinoma, were obtained by endoscopy and treated as above. One carcinoma was studied with regard to possible intratumour heterogeneity by sampling from four different locations.

*Southern blot analysis* Cell suspensions were prepared as described previously (Meling *et al.*, 1993) and stored in 70% ethanol at 4°C until DNA extraction was performed.

### *Probes and labelling*

Northern blot analysis was performed with the entire 2.9 kb human SC cDNA (Krajči *et al.*, 1991) and a *Pst*I fragment from chicken *β*-actin cDNA (Cleveland *et al.*, 1980).

Southern blot analysis was performed with a 5.0 kb *Msp*I fragment from the polymorphic DNA sequence pYNZ23 (locus D1S58) (Nakamura *et al.*, 1987), which exhibits a close linkage centromerically to the SC gene (locus PIGR) (lods + 5.06 at $\Theta_{max} = 0.06$) (Krajči *et al.*, 1992). The probes were labelled with [$\alpha$-$^{32}$P]dCTP (110 TBq $mmol^{-1}$, Amersham, Buckinghamshire, UK) by application of random primers (Feinberg and Vogelstein, 1984).

### *RNA extraction and Northern blot analysis*

Extraction of total RNA and Northern analysis was performed as described previously (Krajci *et al.*, 1989). Autoradiography was accomplished at −70°C, with X-ray film (Hyperfilm-MP Amersham) and intensifying screens (Kodak X-Omatic Super Rapid, Eastman Kodak, NY, USA) for less than 1 day with the *β*-actin probe and for 3–5 days with the SC probe.

### *Densitometric analysis of Northern blot autoradiograms*

Suitably exposed autoradiograms were analysed for optical density (OD) with a 2202 Ultroscan Laser Densitometer (LKB, Bromma, Sweden). For each adenoma and carcinoma

**Table III** RFLP pattern for D1S58 and clinicopathological variables in 32 colorectal carcinomas and peripheral blood mononuclear cells

| *Patient no.* | *Age* | *Sex* | *RFLP*[a] *PBMC*[b] | *Carcinoma* | *Heterozygous informative* | *Allelic loss* | *Grade of differentiation*[c] | *Dukes' stage*[d] | *Bowel location* |
|---|---|---|---|---|---|---|---|---|---|
| 1 | 75 | F | A1A2 | A1 | + | + | Moderate | D | Rectum |
| 2 | 62 | F | A1A2 | A1 | + | + | Moderate | A | Sigmoid colon |
| 3 | 64 | M | A1A2 | A2 | + | + | Moderate | B | Sigmoid colon |
| 4 | 81 | F | A1A2 | A2 | + | + | Poor | B | Caecum |
| 5 | 33 | M | A1A2 | ND[e] | + | +[f] | Poor | C | Rectum |
| 6 | 78 | M | A1A2 | A1A2 | + | − | Well | B | Rectum |
| 7 | 68 | F | A1A2 | A1A2 | + | − | Well | C | Rectum |
| 8 | 63 | M | A1A2 | A1A2 | + | − | Moderate | C | Rectum |
| 9 | 77 | F | A1A2 | A1A2 | + | − | Moderate | B | Caecum |
| 10 | 76 | M | A1A2 | A1A2 | + | − | Moderate | B | Caecum |
| 11 | 79 | F | A1A2 | A1A2 | + | − | Moderate | C | Rectum |
| 12 | 85 | M | A1A2 | A1A2 | + | − | Moderate | B | Rectum |
| 13 | 73 | F | A1A2 | A1A2 | + | − | Moderate | B | Rectum |
| 14 | 61 | M | A1A2 | A1A2 | + | − | Moderate | B | Rectum |
| 15 | 70 | F | A1A2 | A1A2 | + | − | Moderate | B | Right flexure |
| 16 | 74 | F | A1A2 | A1A2 | + | − | Moderate | B | Rectum |
| 17 | 88 | M | A1A2 | A1A2 | + | − | Moderate | B | Sigmoid colon |
| 18 | 65 | F | A1A2 | A1A2 | + | − | Moderate | C | Rectum |
| 19 | 59 | M | A1A2 | A1A2 | + | − | Poor | C | Rectum |
| 20 | 68 | M | A1A2 | A1A2 | + | − | Poor | B | Sigmoid colon |
| 21 | 51 | F | A1A2 | A1A2 | + | − | Poor | B | Rectum |
| 22 | 74 | F | A1A2 | A1A2 | + | − | Poor | C | Rectum |
| 23 | 88 | F | A1A2 | A1A2 | + | − | Poor | C | Right flexure |
| 24 | 65 | F | A1 | A1 | − | − | Well | B | Right flexure |
| 25 | 84 | M | A1 | A1 | − | − | Moderate | C | Ascending colon |
| 26 | 61 | F | A1 | A1 | − | − | Moderate | B | Caecum |
| 27 | 58 | M | A1 | A1 | − | − | Moderate | B | Rectum |
| 28 | 78 | F | A2 | A2 | − | − | Moderate | D | Caecum |
| 29 | 77 | M | A2 | A2 | − | − | Moderate | B | Sigmoid colon |
| 30 | 70 | F | A1 | A1 | − | − | Poor | C | Rectum |
| 31 | 82 | F | A1 | A1 | − | − | Poor | B | Sigmoid colon |
| 32 | 80 | M | A2 | A2 | − | − | Poor | A | Caecum |

[a]Restriction fragment length polymorphism (alleles A1 and A2 are represented by the 5.0 kb and 4.5 kb *Pvu*II fragment on Southern blots respectively). [b]Peripheral blood mononuclear cells. [c]Morson and Sobin (1976). [d]Dukes and Bussey (1958). [e]Not detectable. [f]Diallelic loss.

a value of SC mRNA level was calculated relative to the respective $\beta$-actin mRNA level. Levels of mRNA were assigned a score of 'reduced' and 'increased' relative to the median of the whole sample material.

*Southern blot analysis of RFLP*

Southern blot analysis of *Pvu*II-digested genomic DNA (10 $\mu$g) from colorectal carcinomas and from peripheral white blood cells of the same patients (Meling *et al.*, 1993), was performed as described previously (Krajči *et al.*, 1991). The membranes were exposed to X-ray film with an intensifying screen for 5–8 days at $-70$°C.

*Immunohistochemical staining and evaluation*

The biopsy samples were placed directly from $-70$°C into 96% ethanol at 4°C and further processed for low-temperature paraffin embedding (Brandtzaeg, 1974). One section cut at 6 μm from each tissue block was subjected to direct immunoflourescence staining for 20 h with a fluorescein isothiocyanate (FITC)-labelled sheep anti-SC conjugate (Brandtzaeg, 1981). To control for morphology, an adjacent section was stained by a trichrome routine method with haematoxylin, azofloxine and saffron (Stave and Brandtzaeg, 1977).

Observations were performed by an Aristoplan fluorescence microscope (Leitz, Germany) equipped with an HBO 100 W lamp for excitation of FITC (green) emission. A Ploem-type epi-illuminator was used for narrow-band excitation and filtration. The intensity of epithelial SC fluorescence was scored on an arbitrary semiquantitative scale from 3 (referring to the pattern of normal colonic epithelium) to 0 (indicating virtual lack of staining) (Rognum *et al.*, 1980). Tissue samples with heterogenous staining were scored according to the dominating pattern, the range of scores within the same tumour section being recorded as well.

*Histological grading*

The colorectal adenomas and carcinomas were graded histologically by one observer as showing slight, moderate or severe dysplasia (Jass and Sobin, 1989) and as being well, moderately or poorly differentiated (Morson and Sobin, 1976) respectively. The adenomas with only focal lesions of severe dysplasia were classified together with those showing more extensive severe dysplasia (Table I).

*Statistical analysis*

Expression of SC mRNA, although semiquantitatively determined, was the only truly measured variable in this investigation; fluorescence scores and histological tumour grades were based on subjective ranking. Statistical analyses were therefore adjusted to the limitations given for the ordinary scale (Stevens, 1946) as provided by non-parametric two-tailed rank methods. Group comparisons were based on the Mann-Whitney *U*-test (Siegel, 1956). Epithelial SC staining was grouped in two categories (0–1 and 2–3) for adenomas and (0 and 1–3) for the carcinomas. The histological tumour grade was assigned as 'slight— moderate' or 'severe' for the adenomas and 'slight–moderate' or poor' for the carcinomas. *P*-values of 0.05 or less were considered statistically significant. The sample representing the median value with respect to mRNA expression was not included in the group comparisons.

### Results

*Colorectal adenomas*

*Immunofluorescence staining patterns* The expression of SC was heterogeneous in 20 and homogeneous in 13 adenoma samples (Table I). The staining intensity decreased with increasing grade of dysplasia ($P<0.01$) and SC was undetectable in two cases (Figures 1 and 2a). In general, IgA-positive plasma cells were detected in the vicinity of SC-expressing tumour epithelial cells and the latter often contained IgA as well (data not shown).

*SC mRNA in relation to immunofluorescence staining* - Northern blot analyses demonstrated SC mRNA in variable amounts but with a constant size of approximately 3.8 kb (Figure 3). Reduced SC mRNA levels were noted in 11 of the 12 adenomas that had an SC staining score of 0–1, but only in 4 of those 19 that had a score of 2–3 (Figure 4). This difference was significant ($P\ll0.001$).

*SC mRNA in relation to histological tumour grade* Reduced SC mRNA levels were noted in 11 of the 17 adenomas with severe but in only 5 of 15 tumours with slight–moderate dysplastic changes (Figure 5). However, this trend did not reach significance because of the small number of samples ($P=0.08$).

*Colorectal carcinomas*

*Immunofluorescence staining patterns* SC protein expression was demonstrated in only 9 of the 19 tumours, four with a heterogeneous pattern (Table II). The staining intensity decreased with increasing grade of dysplasia, but this trend did not quite reach significance because of the small number of samples ($P=0.06$).

*SC mRNA in relation to immunofluorescence staining* SC mRNA of normal size was detected in 17 of the tumours. Reduced SC mRNA levels were noted in seven of the ten tumours that had an SC staining score of 0, but only two of them totally lacked the specific message (Table II). Reduced mRNA levels tended to be less common (two cases) among

**Figure 1** Immunofluorescence scores for SC in colorectal adenomas ($n=33$) with different grades of dysplasia collected from 31 patients with gastrointestinal complaints. The broken line connects the median scores for SC protein expression, which was significantly lower in adenomas with severe than with slight–moderate dysplasia.

**Figure 2** Immunofluorescence staining for SC in colorectal neoplasia. (**A**) Adenoma with moderate grade of dysplasia (no. 5, Table I). A heterogenous staining pattern is observed, semiquantitatively scored as 1 and 3 in this part of the tumour. (**B**) Carcinoma, moderately differentiated (no. 10, Table II). A homogenous, negative staining pattern for SC (scored as 0) is observed in this carcinoma (to the right), whereas the positive staining of normal colonic epithelium is shown to the left (scored as 3).

the eight tumours that had an SC staining score of 1–3, although there was no statistically significant difference because of the small number of samples ($P = 0.07$).

The four samples studied from a single carcinoma showed a slightly heterogeneous immunofluoresence staining pattern with intensity scores ranging from 0 to 1, but the mRNA levels appeared to be similar.

*SC mRNA in relation to histological tumour grade* Reduced SC mRNA levels were noted in two of the three carcinomas with poor but in only 7 of 15 tumours with slight–moderate grade of differentiation. No statistical evaluation could be performed because of the small number of poorly differentiated tumours.

*Southern analysis of RFLP and allelic alterations at the D1S58 locus*

*Pvu*II revealed a two-allelic polymorphism for D1S58, namely a 5.0 kb (allele A1) and a 4.5 kb (allele A2) fragment. Twenty-two cases (22 32 = 69%) were heterozygous (informative) for polymorphism on locus D1S58. Heterozygous loss (Figure 6) was demonstrated in four of these tumours (18%). In one additional case (3%) loss of the D1S58 locus was observed on both chromosome 1*q* arms (Table III).

**Figure 3** Northern blot of SC mRNA from six colorectal adenomas collected from six patients with gastrointestinal complaints. Total RNA (10 μg) was extracted, electrophoresed, blotted onto nylon membranes and hybridised with random prime-labelled human SC cDNA probe (top) and chicken β-actin cDNA probe (bottom) (specific activity $2 \times 10^9$ c.p.m. $\mu g^{-1}$ DNA, $10^6$ c.p.m. $ml^{-1}$ hybridisation solution). The patient numbers refer to Table I.

**Figure 4** Scatter diagram of relationship between SC mRNA and SC protein expression in 33 colorectal adenomas collected from 31 patients with gastrointestinal complaints. The broken line connects the median of SC mRNA levels, which was significantly reduced in adenomas with decreased fluorescence score for SC.

### Discussion

This study is the first attempt to analyse the relative SC mRNA expression in colorectal tumours. The increased risk of cancer in adenomas is related to the grade of dysplasia, the tumour size (Morson, 1974), the tendency of bleeding (Doran and Hardcastle, 1982), the tumour number (Matek *et al.*, 1985), the patient's age (The Cancer Registry of Norway, 1982), and the presence of mammary or uterine cancer in the same patient or their first-degree relatives (Giacosa *et al.*,

**Figure 5** SC mRNA expression in 33 colorectal adenomas with different grades of dysplasia collected from 31 patients with gastrointestinal complaints. The broken line connects the median of scores of SC mRNA levels, which tended to be decreased with increasing severity of dysplasia.

**Figure 6** Southern blot analysis of genomic DNA extracted from colorectal carcinomas and distant normal mucosa. Allele changes were detected on chromosome 1*q* by the probe pYNZ23 (specific activity $2 \times 10^9$ c.p.m. $\mu g^{-1}$ DNA, $10^6$ c.p.m. $ml^{-1}$ hybridisation solution) on *Pvu*II-digested blots. Genomic DNA (10 $\mu g$) from normal (N) and tumour (T) tissue of three constitutionally heterozygous patients is shown. Patient 1 is heterozygous for this locus and patient 2 has lost the 5.0 kb allele in the tumour, whereas patient 3 has lost the 4.5 kb allele in the tumour.

1987). Because the present adenoma patients fulfilled such criteria, the examined adenomas (all with a diameter above 10 mm) could be considered as high-risk precancerous lesions.

A significant positive relationship appeared between the SC mRNA levels and the immunofluorescence staining score for SC in the adenomas; this was consistent with the observation that protein expression is generally related to the amount of specific message. The expression of functional SC in the adenomas was supported by the fact that the tumour cells in general showed coexpression for SC and IgA (data not shown). In keeping with previous studies (Isaacson, 1982; Rognum *et al.*, 1982*b*), an inverse relationship existed between the grade of dysplasia and the staining for SC in the adenomas and the same trend was apparent for SC mRNA. SC protein expression in colorectal adenomas might therefore reflect the rate of transcript and/or the stability of specific mRNA.

In the colorectal carcinomas there likewise tended to be a positive relation between SC mRNA and SC staining; however, 53% of the tumours showed very faint or absent SC staining, which could be explained by total lack of SC mRNA in only two specimens. Several possibilities might explain this discrepancy. Firstly, despite an apparently normal RNA size as demonstrated by Northern blot analysis, the SC message in these tumours might be defective in essential translation segments (Munroe and Jacobson, 1990; Falcone and Andrews, 1991) or contain aberrations (frameshift mutations) leading to the synthesis of 'nonsense' protein not recognisable by our polyclonal anti-SC reagent. Such putative mutations could have occurred at the genomic level or during processing of the primary transcript; their detection would need further characterisation of SC mRNA from these tumours, such as cloning and sequencing. Secondly, absent synthesis of SC protein might be due to lack of regulatory factors involved in mRNA translation regulation (Macejak and Sarnow, 1990; Perlmutter, 1990; Ryazanov *et al.*, 1991; Yoon and Donahue, 1992). Thirdly, SC could be subjected to altered post-translational processing (see below).

SC is a specialised transmembrane receptor protein responsible for the translocation of J chain-containing pIgA and pIgM across secretory epithelia (Brandtzaeg and Prydz, 1984). Studies of mutant rabbit SC have demonstrated that the intracytoplasmic segment of SC is essential for its sorting mechanism (reviewed by Mostov, 1994). Altered post-translational modifications, impaired phosphorylation (Casanova *et al.*, 1990) as well as different deletions of this segment result in deviations from the normal trafficking route or cause degradation of SC after endocytosis (Breitfeld *et al.*, 1990). Rognum *et al.* (1982*a*) observed that neoplastic colonic epithelium with moderate or severe dysplasia sometimes contained SC but showed no uptake of IgA, indicating a defect in its pIg receptor function; this might reflect improper post-translational processing of SC during malignant development.

In two carcinomas absent SC protein expression was clearly explained by lack of specific message, which was verified by repeated RNA extractions from parallel tumour samples. Possible reasons for this lack of SC mRNA might be found at the transcriptional level, such as deletions of the SC gene or its regulatory units or absence of protein factor(s) essential for its transcription. Putative deletions would have to involve the SC-encoding gene (locus *PIGR*) on both chromosomes to cause absent message. *PIGR* is located in the 1q31–q41 region (Davidson *et al.*, 1988; Krajči *et al.*, 1991; 1992), which is involved in a large number of recombinantorial events (Brito-Babapulle and Atkin, 1981). Using polymorphic DNA markers, Vogelstein *et al.* (1989) demonstrated that allelic loss on chromosome 1q occurs in approximately 25% of colorectal carcinomas; the corresponding loss of both alleles would then occur at a frequency of about 6%. When we analysed genomic DNA extracted from colonic carcinomas for allelic alterations of locus D1S58, which exhibits a centromeric location of PIGR (Krajči *et al.*, 1992), heterozygous loss of this allele was revealed in 18% and loss of both alleles in 3% of the cases. The estimated frequency of simultaneous loss of both alleles $[(4/22)^2 + 1/32]$ would be more than 6% and might well account for at least one of the two SC mRNA-negative tumour carcinomas. Nevertheless, because the SC mRNA and RFLP analyses were performed on different carcinoma materials, it cannot be excluded that the association between the frequency of SC mRNA loss and the frequency of diallelic loss of locus D1S58 is coincidental.

In conclusion, the positive relationship between mRNA and protein levels of SC observed in colorectal adenomas seemed to be the case also for carcinomas which, however, often lacked detectable SC protein despite expressing some SC mRNA. This difference was remarkable because 55% of the adenomas showed a severe grade of dysplasia. Perhaps cancer SC mRNA contained frameshift mutations and/or was excluded from translation owing to lack of (or suppression by) specific protein factor(s). Deletion of the *PIGR* locus on both chromosomes seemed to be a relatively rare event. The inverse correlation between immunofluorescence staining for SC and grade of dysplasia in the adenomas suggested that reduced SC mRNA expression takes place only late in the carcinogenesis of colorectal neoplasia. A larger tumour sample will have to be analysed to see whether transcription and/or expression of the SC gene might provide information on cellular dedifferentiation during tumour development in the large bowel.

**Acknowledgements**
This work was supported by the Norwegian Cancer Society, the Research Council of Norway, the Legacy of Astrid and Birger Torsted, Anders Jahre's Foundation for the Promotion of Science, The Medical Innovation Foundation at Rikshospitalet, A/S Freia's Medical Fund, and Rakel and Otto Bruun's Legacy. We are grateful for the technical assistance of Tone Narvesen, Bjørg Simonsen and Hanne Malmstrøm.

### References

BRANDTZAEG P. (1974). Mucosal and glandular distribution of immunoglobulin components. Immunohistochemistry with a cold ethanol-fixation technique. *Immunology*, **26**, 1101–1114.

BRANDTZAEG P. (1981). Prolonged incubation time in immunohistochemistry: effects on fluorescence staining of immunoglobulins and epithelial components in ethanol- and formaldehyde-fixed paraffin-embedded tissues. *J. Histochem. Cytochem.*, **29**, 1302–1315.

BRANDTZAEG P. (1985). Role of J chain and secretory component in receptomediated glandular and hepatic transport of immunoglobulins in man. *Scand. J. Immunol.*, **22**, 111–146.

BRANDTZAEG P AND PRYDZ H. (1984). Direct evidence for an integrated function of J chain and secretory component in epithelial transport of immunoglobulin. *Nature*, **311**, 71–73.

BRANDTZAEG P, NILSSEN DE, ROGNUM TO AND THRANE PS. (1991). Ontogeny of the mucosal immune system and IgA deficiency. *Gastroenterol. Clin. N. Am.*, **20**, 397–439.

BRANDTZAEG P, KRAJCI P, LAMM M AND KAETZEL CS. (1994). Epithelial and hepatobiliary transport of IgA. In *Mucosal Immunology, Vol. I: Cellular Basis of Mucosal Immunity*. Ogra PL, Mestecky J, Lamm M, Strober W, McGhee J and Bienenstock J. (eds) pp.113–126. Academic Press: Orlando, FL.

BREITFELD PB, CASANOVA JE, MCKINNON WC AND MOSTOV KE. (1990). Deletions in the cytoplasmic domain of the polymeric immunoglobulin receptor differentially affect endocytotic rate and postendocytotic traffic. *J. Biol. Chem.*, **265**, 13750–13757.

BRITO-BABAPULLE V AND ATKIN NB. (1981). Break points in chromosome #1: abnormalities of 218 human neoplasms. *Cancer Genet. Cytogenet.*, **4**, 215–225.

THE CANCER REGISTRY OF NORWAY. (1982). *Trends in Cancer Incidence in Norway*. 1955–78. The Cancer Registry of Norway: Oslo.

CASANOVA JE, BREITFELD PP, ROSS SA AND MOSTOV KE. (1990). Phosphorylation of the polymeric immunoglobulin receptor required for its efficient transcytosis. *Science*, **248**, 742–745.

CLEVELAND DW, LOPATA MA, MACDONALD J, COWAN NJ, RUTTER WJ AND KIRSCHNER MW. (1980). Number of evolutionary conservation of alpha- and beta-tubulin and cytoplasmic beta- and gamma-actin genes using specific cloned cDNA probes. *Cell*, **20**, 95–105.

CONLEY ME AND DELACROIX DL. (1987). Intravascular and mucosal immunglobulin A: Two separate but related systems of immune defense? *Ann. Int. Med.*, **106**, 892–899.

CORREA P AND HAENSZEL W. (1978). The epidemiology of large-bowel cancer. *Adv. Cancer Res.*, **26**, 1–141.

DAVIDSON MK, LE BEAU MM, EDDY RL, SHOWS TB, DIPIETRO LA, KINGZETTE M AND HANLY WC. (1988). Genetic mapping of the human polymeric immunoglobulin receptor gene to chromo-some region 1q31–q41. *Cytogenet. Cell. Genet.*, **48**, 107–111.

DORAN J AND HARDCASTLE JD. (1982). Bleeding patterns in colorectal cancer: the effect of aspirin and the implications for faecal occult blood testing. *Br. J. Surg.*, **69**, 711–713.

DUKES CE AND BUSSEY HR. (1958). The spread of rectal cancer and its effects on prognosis. *Br. J. Cancer*, **12**, 309–320.

FALCONE D AND ANDREWS DW. (1991). Both the 5′ untranslated region and the sequences surrounding the start site contribute to efficient initiation of translation in vitro. *Mol. Cell Biol.*, **11**, 2656–2664.

FEINBERG AP AND VOGELSTEIN B. (1984). A technique for radiolabeling DNA restriction endonuclease fragments to high specific activity. *Addendum Anal. Biochem.*, **137**, 266–267.

GIACOSA A, SUKKAR SG AND FRASCIO. (1987). The surveillance of high risk patients for colorectal cancer. In *Causation and Prevention of Colorectal Cancer*. Faivre J and Hill M (eds). Elsevier Science Publishers: Amsterdam.

HOFF G, MOEN IE, TRYGG K, FROLICH W, SAUAR J, VATN M, GJONE E AND LARSEN S. (1986). Epidemiology of polyps in the rectum and sigmoid colon. Evaulation of nutritional factors. *Scand. J. Gastroenterol.*, **21**, 199–204.

HOFSTAD B, VATN M, LARSEN S AND OSNES M. (1992). Reliability of in situ measurements of colorectal polyps. *Scand. J. Gastroenterol.*, **27**, 59–64.

ISAACSON P. (1982). Immunoperoxidase study of the secretory immunoglobulin system in colonic neoplasia. *J. Clin. Pathol.*, **34**, 14–25.

JASS JR AND SOBIN LH. (1989). *Histological Typing of Intestinal Tumours*, 2nd edn. World Health Organization, Springer: Geneva.

KORETZ K, SCHLAG P, QUENTMEIER A AND MÖLLER P. (1994). Evaluation of the secretory component as a prognostic variable in colorectal carcinoma. *Int. J. Cancer*, **57**, 365–370.

KRAJCI P, SOLBERG R, SANDBERG M, ØYEN O, JAHNSEN T AND BRANDTZAEG P. (1989). Molecular cloning of the human transmembrane secretory component (poly-Ig receptor) and its mRNA expression in human tissues. *Biochem. Biophys. Res. Commun.*, **158**, 783–789.

KRAJCI P, GRZESCHIK K-H, GEURTZ VAN KESSEL, OLAISEN B AND BRANDTZAEG P. (1991). The human transmembrane secretory component (poly-Ig receptor): molecular cloning, restriction fragment length polymorphism and chromosomal sublocalization. *Hum. Genet.*, **87**, 642–648.

KRAJCI P, GEDDE-DAHL T JR, HØYHEIM B, ROGDE S, OLAISON B AND BRANDTZAEG P. (1992). The gene encoding human transmembrane secretory component (locus PIGR) is linked to D1S58 on chromosome 1. *Hum. Genet.*, **90**, 215–219.

KRAJCI P, TASKÉN K, KVALE D AND BRANDTZAEG P. (1993). Interferon-$\gamma$ stimulation of messenger RNA for human secretory component (poly-Ig receptor) depends on continuous intermediate protein synthesis. *Scand. J. Immunol.*, **37**, 251–256.

KRAKAUER R, ZINNEMAN HH AND HONG R. (1975). Deficiency of secretory Ig-A and intestinal malabsorption. *Am. J. Gastroenterol.*, **64**, 319–323.

KVALE D, BRANDTZAEG P AND LØVHAUG D. (1988). Up-regulation of the expression of secretory component and HLA molecules in a human colonic cell line by tumour necrosis factor-alfa and gamma interferon. *Scand. J. Immunol.*, **28**, 351–357.

MACEJAK DG AND SARNOW P. (1990). Translational regulation of the immunoglobulin heavy-chain binding protein mRNA. *Enzyme*, **44**, 310–319.

MATEK W, GUGGENMOOS-HOLZMANN I AND DEMLING L. (1985). Follow-up of patients with colorectal adenomas. *Endoscopy*, **17**, 175–181.

MELING GI, LOTHE RA, BØRRESEN AL, GRAUE C, HAUGE S, CLAUSEN OP AND ROGNUM TO. (1993). The TP53 tumour suppressor gene in colorectal carcinomas. I. Genetic alterations on chromosome 17. *Br. J. Cancer*, **67**, 88–92.

MORSON BC. (1974). The polyp-cancer sequence in the large bowel. *Proc. R. Soc. Med.*, **67**, 451–457.

MORSON BC AND SOBIN LH. (1976). *International Histological Classification of Tumours*. no 15. World Health Organization: Geneva.

MOSTOV KE. (1994). Transepithelial transport of immunoglobulins. *Annu. Rev. Immunol.*, **12**, 63–84.

MOSTOV KE AND BLOBEL G. (1982). A transmembrane precursor of secretory component. The receptor for transepithelial transport of secretory immunoglobulins. *J. Biol. Chem.*, **257**, 11816–11821.

MUNROE D AND JACOBSON A. (1990). mRNA poly(A) tail, a 3′ enhancer of translational initiation. *Mol. Cell. Biol.*, **10**, 3441–3455.

NAKAMURA Y, CULVER M, O'CONNELL P, LEPPERT M, LATHROP GM, LALOUEL J-M AND WHITE R. (1987). Isolation and mapping of a polymorphic DNA sequence pYNZ23 to chromosome 1 (D1S58). *Nucleic Acids Res.*, **15**, 9620.

NUSSINSON E, LAHAV M, BEREBI A, ESTROV Z, ZUR S AND RESNITZKY P. (1986). Secretory piece and IgA deficiency in a patient with Waldenstrom's macroglobulinemia. *Am. J. Gastroenterol.*, **81**, 995–998.

PERLMUTTER RM. (1990). Translational regulation of the lymphocyte-specific protein tyrosine kinase p56lck. *Enzyme*, **44**, 214–224.

PHILLIPS JO, EVERSON MP, MOLDOVEANU Z, LUE C AND MESTECKY J. (1990). Synergistic effects of IL-4 and IFN-$\gamma$ on the expression of polymeric Ig-receptor (secretory component) and IgA binding to human epithelial cells. *J. Immunol.*, **145**, 1740–1744.

PISKURICH JF, FRANCE JA, TAMER CM, WILLMER CA, KAETZEL CS AND KAETZEL DM. (1993). Interferon-$\gamma$ induces polymeric immunoglobulin receptor mRNA in human intestinal epithelial cells by a protein synthesis dependent mechanism. *Mol. Immunol.*, **30**, 413–421.

PLAUT AG AND RIDKER P. (1992). New light on secretory-component deficiency [letter]. *N. Eng. J. Med.*, **327**, 129.

ROGNUM TO, BRANDTZAEG P, ÖRJASAETER H, ELGJO K AND HOGNESTAD J. (1980). Immunohistochemical study of secretory component, secretory IgA and carcino-embryonic antigen in large bowel carcinomas. *Path. Res. Pract.*, **170**, 126–145.

ROGNUM TO, ELGJO K, FAUSA O AND BRANDTZAEG P. (1982*a*). Immunohistochemical evaluation of carcinoembryonic antigen, secretory component, and epithelial IgA in ulcerative colitis with dysplasia. *Gut*, **23**, 123–133.

ROGNUM TO, FAUSA O AND BRANDTZAEG P. (1982*b*). Immunohistochemical evaluation of carcino-embryonic antigen, secretory component and epithelial IgA in tubular and villous large-bowel adenomas with different grades of dysplasia. *Scand. J. Gastroenterol.*, **7**, 341–338.

RYAZANOV AG, RUDKIN BB AND SPIRIN AS. (1991). Regulation of protein synthesis at the elongation stage. New insights into the control of gene expression in eukaryotes. *FEBS Lett.*, **285**, 170–175.

SCOTT H, BRANDTZAEG P, SOLHEIM BG AND THORSBY E. (1981). Relation between HLA-DR-like antigens and secretory component (SC) in jejunal epithelium of patients with coeliac disease or dermatitis herpetiformis. *Clin. Exp. Immunol.*, **44**, 233–238.

SIEGEL S. (1956). *Non-parametric Statisticas for the Behavioral Sciences*. McGraw-Hill Kogakusha: Tokyo.

SOLLID LM, GAUDERNACK G, MARKUSSEN G, *et al.* (1987). Induction of various HLA class II molecules in a human colonic adenocarcinoma cell line. *Scand. J. Immunol.*, **25**, 175–180.

STAVE R, BRANDTZAEG P. (1977). Fluorescence staining pattern of gastric mucosa. A study with special reference to parietal cells. *Scand. J. Gastroenterol.*, **12**, 885–891.

STEVENS SS. (1946). On the theory of scales of measurement. *Science*, **103**, 677–680.

STROBER W, KRAKAUER R, KLAEVEMAN HL, REYNOLDS HY AND NELSON DY. (1976). Secretory component deficiency. A disorder of the IgA immune system. *N. Engl. J. Med.*, **294**, 351–356.

THRANE PS, SOLLID LM, HAANES HR AND BRANDTZAEG P. (1992). Clustering of IgA-producing immunocytes related to HLA-DR-positive ducts in normal and inflamed salivary glands. *Scand. J. Immunol.*, **35**, 43–51.

VALNES K, BRANDTZAEG P, ELGJO K AND STAVE R. (1984). Specific and nonspecific humoral defense factors in the epithelium of normal and inflamed gastric mucosa. Immunohistochemical localization of immunoglobulins, secretory component, lysozyme, and lactoferrin. *Gastroenterology*, **86**, 402–412.

VOGELSTEIN B, FEARON ER, KERN SE, HAMILTON SR, PREISINGER AC, NAKAMURA Y AND WHITE R. (1989). Allelotype of colorectal carcinomas. *Science*, **244**, 207–211.

YOON H AND DONAHUE TF. (1992). Control of translation initiation in *Saccharomyces cerevisiae*. *Mol. Microbiol.*, **6**, 1413–1419.